# αvβ3 Integrin and Folate-Targeted pH-Sensitive Liposomes with Dual Ligand Modification for Metastatic Breast Cancer Treatment

**DOI:** 10.3390/bioengineering11080800

**Published:** 2024-08-07

**Authors:** Prashant Pandey, Dilip Kumar Arya, Payal Deepak, Daoud Ali, Saud Alarifi, Saurabh Srivastava, Afsaneh Lavasanifar, Paruvathanahalli Siddalingam Rajinikanth

**Affiliations:** 1 Department of Pharmaceutical Sciences, Babasaheb Bhimrao Ambedkar University, Lucknow 226025, Uttar Pradesh, India; 2 Faculty of Pharmacy and Pharmaceutical Sciences, University of Alberta, Edmonton, AB T6G 2E1, Canada; 3 Department of Zoology, College of Science, King Saud University, P.O. Box 2455, Riyadh 11451, Saudi Arabia; 4 Department of Pharmaceutics, National Institute of Pharmaceutical Education and Research, Hyderabad 500037, Telangana, India; 5 Department of Chemical and Material Engineering, University of Alberta, Edmonton, AB T6G 2V4, Canada

**Keywords:** folic acid, iRGD, pH-sensitive liposomes, breast cancer, cytotoxicity

## Abstract

The advent of pH-sensitive liposomes (pHLips) has opened new opportunities for the improved and targeted delivery of antitumor drugs as well as gene therapeutics. Comprising fusogenic dioleylphosphatidylethanolamine (DOPE) and cholesteryl hemisuccinate (CHEMS), these nanosystems harness the acidification in the tumor microenvironment and endosomes to deliver drugs effectively. pH-responsive liposomes that are internalized through endocytosis encounter mildly acidic pH in the endosomes and thereafter fuse or destabilize the endosomal membrane, leading to subsequent cargo release into the cytoplasm. The extracellular tumor matrix also presents a slightly acidic environment that can lead to the enhanced drug release and improved targeting capabilities of the nano-delivery system. Recent studies have shown that folic acid (FA) and iRGD-coated nanocarriers, including pH-sensitive liposomes, can preferentially accumulate and deliver drugs to breast tumors that overexpress folate receptors and αvβ3 and αvβ5 integrins. This study focuses on the development and characterization of 5-Fluorouracil (5-FU)-loaded FA and iRGD surface-modified pHLips (FA-iRGD-5-FU-pHLips). The novelty of this research lies in the dual targeting mechanism utilizing FA and iRGD peptides, combined with the pH-sensitive properties of the liposomes, to enhance selective targeting and uptake by cancer cells and effective drug release in the acidic tumor environment. The prepared liposomes were small, with an average diameter of 152 ± 3.27 nm, uniform, and unilamellar, demonstrating efficient 5-FU encapsulation (93.1 ± 2.58%). Despite surface functionalization, the liposomes maintained their pH sensitivity and a neutral zeta potential, which also conferred stability and reduced aggregation. Effective pH responsiveness was demonstrated by the observation of enhanced drug release at pH 5.5 compared to physiological pH 7.4. (84.47% versus 46.41% release at pH 5.5 versus pH 7.4, respectively, in 72 h). The formulations exhibited stability for six months and were stable when subjected to simulated biological settings. Blood compatibility and cytotoxicity studies on MDA-MB-231 and SK-BR3 breast cancer cell lines revealed an enhanced cytotoxicity of the liposomal formulation that was modified with FA and iRGD compared to free 5-FU and minimal hemolysis. Collectively, these findings support the potential of FA and iRGD surface-camouflaged, pH-sensitive liposomes as a promising drug delivery strategy for breast cancer treatment.

## 1. Introduction

Breast cancer (BC) stands as the most frequently diagnosed cancer and the second primary contributor to cancer-related fatalities in women globally [1]. According to the American Cancer Society’s estimates, over 310,720 new cases of invasive BC are expected to be detected in the United States in 2024, along with 55,500 new cases of ductal carcinoma (in situ) BC [2]. Additionally, it is predicted that 42,250 deaths from BC will occur in the US in 2024 [2]. 5-Fluorouracil (5-FU) is a pyrimidine analog chemotherapy drug commonly used to treat BC [3]. The mechanism of action of 5-FU involves inhibiting the synthesis of thymidylate, an essential component of DNA, by targeting the enzyme thymidylate synthase. This leads to DNA damage and, eventually, apoptosis in cancer cells [4]. However, this effect can also be seen in other healthy, proliferating cells. In addition, 5-FU treatment also leads to the formation of reactive oxygen species (ROS) and the induction of apoptosis or other side effects in healthy cells. The non-specific mechanism of action of 5-FU can lead to a range of side effects, including gastrointestinal toxicity, myelosuppression, and hand–foot syndrome in patients [5]. Poly(lactide-co-glycolide) (PLGA) nanocomposite particles incorporating both 5-FU and gold-decorated magnetite nanoparticles with a raspberry-like morphology have been developed as versatile tools in drug delivery systems and cancer therapy. This innovative formulation offers enhanced dual magneto- and photothermal responses due to the presence of both gold and Fe_3_O_4_ nanoparticles. The controlled drug release profile of these nanocomposite particles, observed at 37 °C in phosphate-buffered saline solution, underscores their potential in cancer therapy. Specifically, their ability to release 5-FU and respond to photothermal stimuli highlights their efficacy in treating DU145 prostate cancer cells. This dual functionality not only enhances therapeutic performance but also aims to minimize side effects, marking a significant advancement in multidisciplinary approaches to cancer treatment [6,7].

The utilization of liposomes for drug delivery has shown to be an effective strategy to improve the solubility of poorly soluble drugs or to enhance the delivery of drugs to tissue and cellular targets in the biological system [8]. Liposomes are favored for drug delivery due to their compatibility with biological systems and ability to encapsulate a wide range of molecules. Hydrophilic molecules are trapped in the aqueous core, while hydrophobic molecules are housed within the lipid bilayer. The lipid composition affects liposome characteristics, including size, surface charge, rigidity, and drug release kinetics [9,10,11]. For instance, compared to unsaturated phosphatidylcholine species, saturated phospholipids with longer acyl chains, such as 1,2-dipalmitoyl-sn-glycero-3-phosphocholine (DPPC), produce a more rigid and durable bilayer structure [12].

For long circulation, liposomes are commonly made using 1,2-distearoyl-sn-glycero-3-phosphoethanolamine-N-amino-polyethyleneglycol-2000 (DSPE-mPEG-_2000_) [13]. The gold standard long-circulating liposomal formulation taking advantage of DSPE-PEG is the liposomal formulation of doxorubicin, i.e., Doxil^®^. This formulation of doxorubicin is shown to lessen the drug-related cardiotoxicity and gastrointestinal adverse effects like nausea and vomiting in clinic [14]. Liposomal formulation is suggested to achieve this by reducing anthracycline delivery to heart tissue and the tissues of the gastrointestinal tract. Despite all advantages, liposomal drug delivery encounters notable challenges, such as delayed drug release in the target tissue and the potential for inefficient cellular drug delivery due to a deficiency in fusogenic behavior after cellular internalization in the endosomal compartment [15,16].

The advancement of pH-responsive liposomes offers a hopeful approach in overcoming these challenges, particularly for cancer therapy. This approach exploits the acidic characteristic of tumors which exhibit lower pH values than healthy tissues [17]. Tumor tissues exhibit an acidic extracellular pH due to factors like anaerobic glycolysis and increased CO_2_ production, resulting in pH levels ranging from 5.5 to 7.0, significantly lower than physiological pH [18,19,20]. Stimuli-responsive liposomes can be designed to selectively release medications within the acidic tumor microenvironment [21]. These pH-sensitive liposomes remain stable under normal physiological pH conditions but become destabilized, leading to drug release, when exposed to the acidic microenvironment of tumors [22].

Dioleoylphosphatidylethanolamine (DOPE) is a crucial element in the composition of pH-sensitive liposomes [23]. When liposomes containing DOPE encounter an acidic environment, they undergo destabilization. This behavior is primarily attributed to the reduced hydration of DOPE’s polar head group, resulting in a hexagonal inverted phase and the formation of non-lamellar structures that induce the destabilization of liposome bilayers in acidic conditions [23,24]. This unique property allows the liposomes to release their cargo specifically in response to the acidic pH of the tumor microenvironment, while maintaining their stability in plasma. This capability can also significantly enhance the delivery of various agents into the cytoplasm upon the liposomes’ internalization into the acidic endosomal environment. The utilization of non-bilayer lipids, like DOPE, which possesses a cone-shaped structure and cannot independently form a bilayer, can be stabilized in a bilayer configuration through the addition of a stabilizing lipid, such as phosphatidylserine or a weakly acidic amphiphile like cholesteryl hemisuccinate (CHEMS) [25]. The inclusion of a stabilizing lipid ensures the liposomes’ stability under neutral pH conditions. However, in an acidic environment, the stabilizing lipid undergoes partial protonation, diminishing its stabilizing influence on the bilayer structure. This reduction in stabilizing effects allows DOPE molecules to transition to their inverted hexagonal phase, disrupting liposomal bilayer organization and leading to the subsequent release of the payload. This innovative approach has been employed for targeted drug delivery to cancer cells within an acidic tumor microenvironment [22].

To enhance the selective uptake of liposomes by cancer cells, researchers have incorporated various ligands onto the surface of liposomes based on the overexpression of specific receptors on the surface of cancer cells [26,27,28]. Folate receptors are overexpressed in more than 40% of tumors, including BC. Therefore, coating liposomes with folic acid has emerged as a promising approach to improve their uptake by cancer cells while reducing uptake by normal cells [29,30]. Gazzano et al. demonstrated an innovative approach using folate receptor-targeted liposomes loaded with doxorubicin (DOX) conjugated to nitric oxide (NO)-releasing groups to combat P-glycoprotein (P-gp) drug efflux in multidrug-resistant (MDR) BC [31]. FA-coated liposomes facilitated targeted delivery, localizing to the nucleus and mitochondria, where DOX-induced DNA damage, cell cycle arrest, and mitochondria-dependent apoptosis [32]. In mouse models, this system effectively suppressed breast tumor growth with P-gp and folate receptors, outperforming free DOX and Caelyx^®^. The treatment maintained its efficacy and potential resistance prevention across multiple cycles. In another study, Barbosa et al. developed pH-sensitive, targeted liposomes using CHEMS and lipid-PEG-folate for paclitaxel (PTX) delivery in metastatic breast cancer. The addition of DOPE enhanced the pH sensitivity by promoting membrane fusion in acidic environments. Viability studies showed that folate-targeted, pH-sensitive liposomes were more cytotoxic than non-targeted versions or free PTX, especially in MDA-MB-231 cells, which have higher folate receptor levels [33]. In addition to folate receptors, a peptide called iRGD has been investigated as a targeting ligand on liposomes to improve their endocytosis into cancer cells and, at the same time, enhance liposomal tumor penetration. iRGD can selectively bind to the αvβ3 integrins that are highly expressed on the surface of tumor endothelial cells and cancer cells. Once bound to the integrins, iRGD also binds to neuropilin-1 (NRP-1) and triggers a process known as the tumor-penetrating peptide (TPP) effect, which facilitates deep penetration of the liposomes into the tumor tissue. Therefore, iRGD is considered a promising ligand for enhancing the selective delivery of drugs to the tumor microenvironment by promoting the endocytosis of liposomes into cancer cells and improving their penetration within the tumor [34,35]. Despite the advancements in liposomal drug delivery systems, significant challenges remain, particularly in achieving efficient drug release and selective targeting within the tumor microenvironment. The current research introduces a novel dual-targeting liposomal system that leverages both FA and iRGD peptides for enhanced specificity and efficacy in drug delivery to breast cancer cells. This dual modification is combined with pH-sensitive liposomes (pHLips), creating a formulation (FA-iRGD-5-FU-pHLip) that is not only responsive to the acidic tumor microenvironment but also exhibits targeted delivery through receptor-mediated endocytosis. This combination has not been previously explored in such a detailed and systematic manner for 5-FU delivery. The novelty lies in the integration of dual-targeting ligands with pH-sensitive liposomes, which is expected to improve the selective targeting and uptake by cancer cells and effective drug release in the acidic tumor environment. This advancement addresses the significant limitations of current chemotherapeutic approaches, potentially reducing side effects and improving therapeutic efficacy.

## 2. Experimental Section

### 2.1. Materials

5-Fluorouracil (5-FU) was purchased from HiMedia (Mumbai, India). Phospholipids with 80% phosphatidylcholine (E80) were obtained as a generous gift sample from Lipoid GmbH (Ludwigshafen, Germany). Cholesteryl hemisuccinate (CHEMS) and Dioleylphosphatidylethanolamine (DOPE) were procured from ChemScence (Burlington, MA, USA). 1-Ethyl-3-[3-dimethylaminopropyl] carbodiimide hydrochloride (EDC), sulfo-N-hydroxy succinimide (sulfoNHS), and Triton X-100 were purchased from Sigma-Aldrich (Burlington, MA, USA). 1,2-distearoylsn-glycero-3-phosphoethanol amine-N- [carboxy (polyethylene glycol)-2000] (Na^+^ salt) (DSPE-PEG_2000_-COOH) was purchased from Avanti Polar Lipids, Inc. (Alabaster, AL, USA).

MTT (3-(4,5-dimethylthiazol-2-yl)-2,5-diphenyl tetrazolium bromide) dye, DAPI (4′,6-diamidino-2-phenylindole, dihydrochloride) dye, and FITC (fluorescein isothiocyanate) dye were obtained from Sigma-Aldrich, St. Louis, MO, USA. Dubelcco’s Modified Eagle’s Medium (DMEM) culture medium, antibiotic PSA (penicillin, streptomycin, and amphotericin B), and trypsin were purchased from Invitrogen (Thermo Fisher Scientific—Sao Paulo, Brazil). Fetal bovine serum (FBS) was acquired from Gibco (Sao Paulo, Brazil). A dialysis membrane (MWCO 12,500–14,000 Da) was purchased from Himedia, Mumbai. Membrane filters (0.2 μm) and all other analytical-grade chemicals were obtained from Thermo Fisher. All other reagents and chemicals acquired were of analytical grade. MDA-MB-231 and SK-BR-3 cell lines were obtained from the National Centre for Cell Science (NCCS), Pune, India.

### 2.2. Methodology

#### 2.2.1. Experimental Design and Optimization of 5-FU-pHLip

Response surface methodology (RSM) was employed as a design of experiments (DoE) technique to build and analyze second-order polynomial models. This was done throughout 15 experiment runs. The optimization process was conducted using Design-Expert^®^ software (Version 13.0, Stat-Ease Inc., Minneapolis, MN, USA). A 3-factor, 3-level Box-Behnken design (BBD) was employed to investigate the influence of independent variables, namely the molar ratio of E80: DOPE (X1), molar concentration of CHEMS (X2), and drug amount (X3), on dependent variables including particle size (PS) (Y1), % entrapment efficiency (EE) (Y2), and % pH sensitivity (Y3). The independent variable was manipulated at three levels (−1, 0, and +1), representing low, medium, and high values, respectively.

Table 1 presents specific information about the independent and dependent variables that were considered during the experimental design, including their coded and decoded values. The best model was determined by computing and comparing several parameters, such as the multiple correlation coefficient (R2), adjusted multiple correlation coefficient (adjusted R2), coefficient of variation (CV), and the anticipated residual sum of squares. In addition, Design-Expert^®^ software (version 13.0, Stat-Ease Inc., Minneapolis, MN, USA) was used to create 3D response surface plots and 2D contour plots.

#### 2.2.2. Synthesis of DSPE-PEG_2000_-FA Conjugate

DSPE-PEG_2000_-FA was synthesized according to a previously outlined protocol [36]. In brief, 33.08 mg of FA with a concentration of 0.15 mM was dissolved in dimethyl sulfoxide (DMSO). N-hydroxysuccinimide (NHS) (10.36 mg) and 1-(3-dimethylaminopropyl)-3-ethylcarbodiimide hydrochloride (EDCl) (17.25 mg) were added to the mixture. The mixture was allowed to stand at room temperature for 30 min. Afterward, 100 mg of DSPE-PEG_2000_-amine dissolved in DMSO was added to the reaction mixture. Following this, the resulting supernatant underwent dialysis using a membrane with a molecular weight cutoff of 10 kDa. Dialysis was carried out sequentially against saline twice and water thrice to eliminate residual DMSO and unconjugated reactants. Confirmation of the DSPE-PEG_2000_-FA conjugate formation was achieved through Fourier Transform Infrared Spectroscopy (FTIR) (Nicole 6700, Thermo Scientific, Waltham, MA, USA) and ^1^H NMR analyses (500 MHz, JEOL, Peabody, MA, USA).

#### 2.2.3. Synthesis of the DSPE-PEG_2000_-iRGD Conjugate

The DSPE-PEG_2000_-iRGD conjugate was synthesized using a previously reported procedure with a few modifications [37]. Briefly, DSPE-PEG_2000_-maleimide ammonium salt (MW 2941.61) and iRGD peptide were dissolved individually in a pH 8 buffer solution containing 50 mM triethanolamine hydrochloride, 50 mM sodium phosphate, 150 mM NaCl, and 1 mM EDTA. Subsequently, the DSPE-PEG_2000_-maleimide solution (2 mM) was carefully combined with an equivalent amount of iRGD peptide solution (4 mM) and allowed to react overnight in a glass container at a temperature of 4 °C. The synthesized product was confirmed using ^1^H NMR spectroscopy.

#### 2.2.4. Preparation of Plain 5-FU-Loaded, pH-Sensitive Liposomes (5-FU-pHLips) and Dual Ligand Surface-Modified, 5-FU-Loaded, pH-Sensitive Liposomes (FA-iRGD-5-FU-pHLips)

This study developed pH-sensitive liposomes through a previously reported thin film hydration method using an E-80/DOPE/CHEMS/DSPE-PEG_2000_ mixture according to the required molar ratio/concentration. Briefly, lipids and 5-FU were dissolved in a chloroform–methanol mixture (9:1) and then transferred to a rotating flask to evaporate the organic solvent under reduced pressure to form a thin film. Afterward, NaOH solution (CHEMS/NaOH ratio 1:1) was added and shaken vigorously to induce the complete ionization of CHEMS and form a multilayer structure. Liposomes were sonicated at 40% amplitude for 2 min in an ice bath using a probe sonicator (PRO900, Labman, scientific instruments, Chennai, India). The free drug was removed by centrifugation at 4000 rpm for 10 min at 4 °C, and the liposomal formulations were stored at 4 °C. Likewise, dual ligand surface-camouflaged, pH-sensitive liposomes were also prepared in the same manner as above, by adding DSPE-PEG_2000_-FA and DSPE-PEG_2000_-iRGD conjugates (1:1 ratio) to lipids at the required molar ratio. Finally, the prepared liposomal formulations were stored at 4 °C and characterized by an FTIR analysis [38].

#### 2.2.5. Physicochemical Characterization of the Prepared pHLips’

##### Particle Size (PS), Polydispersity Index (PDI), and Zeta Potential (ZP)

The average size of the vesicles and their polydispersity index (PDI) were determined using dynamic light scattering (DLS) at a temperature of 25 °C and a scattering angle of 90°. In addition, electrophoretic mobility, combined with DLS, was used to measure the zeta potential (ZP). The tests were conducted using a Nano zetasizer (NSZ90, Malvern Instruments, Worcestershire, UK). Prior to analysis, all samples were diluted in a 0.9% (*w*/*v*) NaCl solution at a 1:100 ratio. To ensure precision, each measurement was conducted in triplicate. The vesicle size and its size distribution were determined by calculating the average diameter (hydrodynamic diameter (D_H_)) using the Gaussian distribution function [39].

##### Surface Morphological Analyses by Transmission Electron Microscopy (TEM) and Atomic Force Microscopy (AFM)

TEM images of the FA-iRGD-5-FU-pHLip were obtained using a negative staining technique to complement the morphological evaluation. Initially, the liposomal formulations were diluted and applied onto a carbon film. Subsequently, the samples were placed onto a formvar-coated copper grid and stained with a solution consisting of 2% (*w*/*v*) phosphotungstic acid, 0.5% (*w*/*v*) bovine serum albumin, and 0.5% (*w*/*v*) saccharose to serve as a contrast agent. The stained samples were then observed at ambient temperature and various voltages using a TECNAI 200 Kv (FEI Electron Optics, Eindhoven, The Netherlands) microscope.

Furthermore, the surface morphology of the FA-iRGD-5-FU-pHLip was also determined using AFM. About 30 μL of the diluted formulation was placed on a mica surface for an AFM analysis. After drying at ambient temperature, samples were analyzed by AFM microscopy, NTEGRA Prima AFM (NT-MDT, Moscow, Russia), in contact mode [40].

##### Percent Entrapment Efficiency and Drug Loading

The % EE and % DL of the developed 5-FU-pHLip and FA-iRGD-5-FU-pHLip were assessed using the high-performance liquid chromatography (HPLC) method [41]. To do this, a predetermined volume of liposomal solution (500 µL) was centrifuged at 2000 rpm for 5 min using Nanosep^®^ (Pall Corp., Exton, PA, USA) advanced centrifugal tubes in order to separate the unentrapped drug. A 1% solution of Triton X-100 was used to disrupt the bilayer of the liposomes and subsequently passed through a 0.22 µm filter. Afterward, the solution was diluted by adding a mobile phase consisting of a mixture of water and methanol in a ratio of 98:02 (*v*/*v*). The diluted solution was then injected into a C18 column (250 mm × 4.6 mm i.d., with 5 µm) (Shimadzu, Corp., Kyoto, Japan). The elution process lasted 5 min, with a 1 mL/min flow rate. Detection was conducted using a UV detector (Shimadzu Prominence-i LC-2030 Plus, Shimadzu, Corp., Kyoto, Japan) set at a wavelength of 266 nm [42,43]. The values for % EE and % DL were determined using the following equation.

$$\%\;EE=\frac{Weight\;of\;entrapped\;drug}{Initial\;weight\;of\;drug\;added\;}\times 100$$

$$\%\;DL=\frac{Weight\;\;of\;entrapped\;\;drug}{Weight\;of\;entrapped\;drug+Weight\;lipids\;used\;in\;the\;liposomes\;preparation}\times 100$$

##### In-Vitro pH Sensitivity

The pH sensitivity of the liposomal formulations (5-FU-pHLip and FA-iRGD-5-FU-pHLip) was assessed employing the dialysis bag method. Briefly, dialysis bags with a molecular weight cutoff of 12 kDa were filled with 5 mL of liposomal suspension. These bags were then incubated in two different release media: PBS (pH 7.4)–methanol (7:3) and PBS (pH 5.0)–methanol (7:3). Both release media contained 0.1% (*v*/*v*) Tween 80 to ensure that there was a sufficient amount of liposomal suspension available for diffusion. The dialysis bags were placed in flasks and agitated on an orbital shaker at 200 rpm, at 37 ± 2 °C. At specified time intervals (0, 1, 2, 4, 6, 8, 10, 12, 24, 48, and 72 h), 0.5 mL samples were taken from the release media and replaced with an equivalent volume of fresh medium. The quantity of 5-FU released was quantified using the previously reported HPLC method. The percentage cumulative release of 5-FU from the liposomal formulations was calculated using the following equation:

$$Percentage\;Cumulative\;Release\;\left(\%\right)=\left(\frac{C_{i}+\sum_{i=1}^{n-1}C_{i}\times V_{i}}{C_{total}}\right)\times 100$$

where C_i_ is the concentration of 5-FU in the previous samples, V_i_ is the volume of the release medium, and C_total_ is the initial concentration of 5-FU in the liposomal suspension.

#### 2.2.6. pH-Induced Liposomal Aggregation and Change in Zeta Potential (ZP)

The liposomal aggregation induced by alterations to the pH was assessed by measuring the increase in particle size. The liposomal suspensions were diluted using acetate buffers with varying pH levels and then subjected to incubation at 37 ± 2 °C. The liposomes’ dispersions were measured thrice at 37 ± 2 °C in 20 mM acetate buffer (pH 5.5 and 6.5) or 20 mM PBS (pH 7.4). At different time periods, aliquots of the samples were taken and the hydrodynamic diameter (D_H_) was measured using a Zetasizer. In addition, we also evaluated any changes in the ZP of the liposomes that were incubated at different pH values [41].

#### 2.2.7. Storage Stability Study

In order to evaluate the shelf life of the liposomal formulation, the developed FA-iRGD-5-FU-pHLip was exposed to two different temperature conditions: refrigerator storage (4 ± 2 °C) and room temperature storage (25 ± 2 °C, 65% relative humidity). For this study, aliquots were collected at specified intervals of 6 months. Measurements of particle size (D_H_) and zeta potential were conducted as previously described. Concurrently, aliquots were collected at each time point to determine the particle size, zeta potential, and percentage of drug entrapment efficiency [44].

#### 2.2.8. In-Vitro Biological Stability

The liposomal suspension of the developed FA-iRGD-5-FU-pHLips was mixed separately with PBS (pH 7.4) and DMEM culture media. The media were supplemented with 5% (*v*/*v*) FBS. This was done to assess the behavior of the liposomes in biological assays, including both in-vitro and in-vivo studies. The liposome was diluted in PBS or DMEM at a volume ratio of 1:4, and then incubated at 37 °C for 24 h. The samples were collected before incubation and after 24 h to evaluate the size of particles (D_H_) and their zeta potential using the methods described before.

#### 2.2.9. Serum Stability Assessment

The stability of the pHLip formulations in serum was tested by substituting buffers with 80% serum (prepared in PBS pH 7.4) taken from healthy Wistar rats. Changes in particle size (D_H_) and zeta potential were then measured after incubating the preparations at 37 °C for 48 h. The samples were analyzed for particle size and zeta potential using the abovementioned methods.

#### 2.2.10. Hemolysis Study

The hemolytic investigation utilized blood samples extracted from healthy female Wistar rats via a retro-orbital plexus puncture and collected in EDTA (0.2%) coated microcentrifuge tubes. After centrifugation at 5000 rpm for 5 min, the red blood cells (RBCs) and plasma were separated and washed with normal saline (0.9% *w*/*v* NaCl solution) until a clear, colorless supernatant was achieved. The washed cells were subsequently reconstituted in 5 mL of normal saline, resulting in a 10% *w*/*v* hematocrit solution. Then, 1 mL aliquots of the hematocrit solution were dispensed into individual tubes and combined with the formulations to ensure uniform drug and formulation concentrations across all samples. After gentle shaking and a 30-min incubation period, the tubes underwent centrifugation at 5000 rpm for 5 min. The resulting supernatant was then diluted using Triton X-100 and normal saline in equal proportion. At 540 nm, the absorbance was then measured. As positive and negative controls, respectively, there was Triton X-100 and normal saline [45]. The hemolysis percentage was determined using the formula provided below.

$$Hemolysis\;\left(\%\right)=\frac{OD\;\left(test\right)-OD\;\left(negative\;control\right)}{OD\;\left(positive\;control\right)-OD\;\left(negative\;control\right)}\times 100$$

where, OD symbolizes the mean optical density and test denotes the supernatant of the prepared samples (RBCs along with 5-FU/5-FU-pHLip/FA-iRGD-5-FU-pHLip).

#### 2.2.11. In-Vitro Cell Lines Studies

##### MTT Assay for Cell Cytotoxicity Study

The MTT assay was employed to evaluate the cytotoxicity of free 5-FU and the developed formulations (5-FU-pHLip and FA-iRGD-5-FU-pHLip) in MDA-MB-231 and SK-BR3 BC cell lines for 48 and 72 h. Initially, 100 µL of cell suspension, containing a density of 2 × 10^4^ cells/well, was seeded into 96-well, flat-bottomed tissue culture plates and incubated at 37 °C in a 5% CO_2_ incubator for 24 h. Simultaneously, various dilutions of the pure drug and developed formulation concentrations were prepared using culture media, and 100 µL/well was added in triplicate to the plates, followed by a further 24-h incubation period. Control wells were preserved by adding an identical proportion of cells in the growth medium without any drugs. Afterward, 20 µL of MTT solution at a concentration of 5 mg/mL was added to each well and left to incubate for a duration of 2 h. After a duration of 2 h, the non-reduced MTT and medium were withdrawn. In addition, the purple, needle-shaped formazan crystals generated by the MTT dye were dissolved using 100 µL of DMSO. Subsequently, the plates were agitated for a duration of 20 min, and the optical density was quantified at a wavelength of 590 nm using a microplate reader. All tests were conducted in triplicate [46].

##### Wound Healing Study by Scratch Assay

In order to assess wound healing, MDA-MB-231 and SK-BR3 tumor cells were placed in 12-well plates at a concentration of 3 × 10^5^ cells per well and were allowed to form a monolayer by incubation at a temperature of 37 °C for 24 h. Afterward, a linear scratch was created in each well (n = 3) utilizing a 10 µL pipette tip, and photos were taken with a microscope to establish the control. After creating the scratch, the wells were treated with 1 mL of new DMEM solution containing 1% (*v*/*v*) FBS, which was supplemented with either the drug or liposomal formulations. The plates were subsequently placed in an incubator at a temperature of 37 °C for an extra duration of 24 h. This method was implemented to reduce inconsistency among wells by capturing images of identical scratches and computing the wound size both before and after the treatment. The extent of the wounds was measured using the MRI Wound Healing Tool plugin, a feature of the ImageJ 1.45 software (National Institutes of Health, Bethesda, MD, USA) [47,48]. The % wound healing was determined using the equation.

$$Wound\;closure\;\left(\%\right)=\left(\frac{Initial\;wound\;area-Final\;wound\;area}{Initial\;wound\;area}\right)\times 100$$

### 2.3. Statistical Analysis

The statistical analysis was conducted using GraphPad Prism (version 8.0) software. The numerical data were produced using the mean ± SD. The data were analyzed through one-way and two-way ANOVA. Subsequently, Dunnett and Tukey’s multiple comparison tests were conducted. A *p*-value of less than 0.05 was considered to show significant differences, while a *p*-value of less than 0.01 was considered highly significant.

## 3. Result and Discussion

### 3.1. Box-Behnken Design (BBD) Analysis

We utilized response surface methodology to investigate the impact of independent variables, including the molar ratio of E80:DOPE (A), the molar concentration of CHEMS (B), and the amount of drug (C), on dependent variables, such as particle size (Y1), % entrapment efficiency (Y2), and % pH sensitivity (Y3).

A total of 15 experimental runs were conducted for this investigation, and the outcomes of the responses are displayed in Table 2. A quadratic model was determined by fitting the values of the dependent variables acquired for multiple trial formulations into the model environment, resulting in the most accurate model. A polynomial equation including the primary influence and interaction factors has been altered to illustrate the impact of each factor on the responses. A plus (+) or minus (−) sign indicates each factor’s impact on the responses.

#### 3.1.1. Impact of an Independent Variable on Particle Size (PS) (Response Y1)

**Figure 1:**
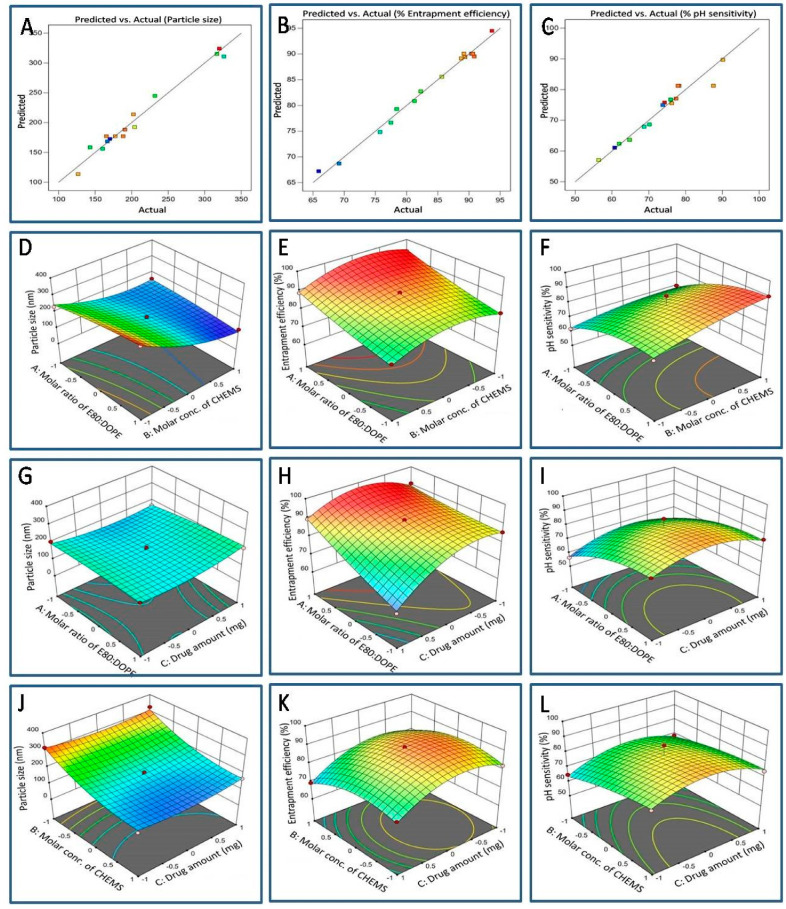
Panels (**A**–**C**) depict linear correlation plots illustrating the relationship between predicted and actual values for particle size, % entrapment efficiency, and % pH sensitivity, respectively. Panels (**D**–**F**) display surface response 3D plots demonstrating the impact of the molar ratio of E80:DOPE and molar concentration of CHEMS on particle size, % entrapment efficiency, and % pH sensitivity, respectively. Panels (**G**–**I**) show surface response 3D plots illustrating the impact of the molar ratio of E80:DOPE and the amount of drug on particle size, % entrapment efficiency, and % pH sensitivity, respectively. Panels (**J**–**L**) display surface response 3D plots showcasing the effect of the molar concentration of CHEMS and the amount of drug on particle size, % entrapment efficiency, and % pH sensitivity, respectively.

In nanoformulation, the PS is crucial in influencing biodistribution and cellular uptake. The impact of each independent variable on the PS of the developed 5-FU-pHLip formulation is illustrated in Figure 1D–F and Table 2. The PS of the developed formulation varied from 126.7 ± 1.6 nm to 326.4 ± 1.2 nm across the different formulations that were prepared using various combinations of factors.

The results of the one-way ANOVA for the quadratic model indicate a significant effect of the molar ratio of E80 and DOPE, molar concentration of CHEMS, and drug amount on PS, with a *p*-value of 0.0015 (*p* < 0.05). The model F-value of 22.85 suggests that the model is statistically significant, with only a 0.15% chance that such a large F-value could occur due to random variation. Additionally, the lack of fit F-value for PS particle size was found to be 2.85, indicating that the lack of fit is insignificant relative to the pure error (*p* > 0.05). There is a 27.08% chance that a lack of fit F-value this large could occur due to random variation.

In fit statistics, the adjusted R^2^ and predicted R^2^ values were found to be 0.9598 and 0.8848, respectively, which ensured the difference was <0.2. The minimal difference (less than 0.2) between the predicted R^2^ (0.9911) and adjusted R^2^ (0.9905) values indicated the adequacy of the selected model for the prediction of responses. It confirms that the model can be used to navigate the design space.

The polynomial regression equation generated by the RSM for particle size with respect to factors A, B, and C is as follows:

PS = +177.22 + 9.22* A − 74.78* B + 1.38* C − 30.48* AB + 11.37* AC + 3.56* BC − 6.94* A^2^ + 39.48* B^2^ + 21.64* C^2^

where A is the molar ratio of E80 and DOPE, B is the molar concentration of CHEMS, and C is the amount of drug.

For the quadratic equation provided, the plus (+) and minus (−) signs signify the direction of the change in the response with respect to the factors. Only factor B was deemed significant among the chosen independent variables and their interactions (*p* < 0.0001), indicating a substantial effect of coefficient B on the liposome’s particle size. Figure 1D–F illustrates the 3D surface response plot for particle size. It shows that decreasing the molar ratio of E80 and DOPE and increasing the molar concentration of CHEMS results in a more significant decrease in particle size. On the other hand, increasing the molar ratio of E80 and DOPE, as well as the amount of drug, leads to a significant enlargement in particle size. Moreover, the particle size achieved at higher molar concentrations of CHEMS is smaller compared to that achieved at lower molar concentrations of CHEMS. This might be related to the fact that CHEMS enhances the fluidity of the bilayer membrane.

#### 3.1.2. Impact of Independent Variables on EE (Response Y2)

The influence of various 5-FU-pHLip variables on % entrapment efficiency is evident in Figure 1G–I and Table 2. The % EE of the developed 5-FU-pHLip batches ranged from 65.91 ± 2.18% to 93.67 ± 1.18%. The results obtained from the one-way ANOVA for the quadratic model revealed a significant effect of the molar ratio of E80 and DOPE, molar concentration of CHEMS, and drug amount on % entrapment efficiency with a *p*-value of 0.0001 (*p*-value < 0.05). The ANOVA for the quadratic model yielded a model F-value of 65.65, indicating the model’s significance, with only a 0.001% chance of such a large F-value occurring due to noise. Additionally, the lack of fit F-value was found to be 4.13, suggesting that the lack of fit is insignificant relative to the pure error (*p*-value > 0.05). There is only a 0.11% chance that a lack of fit F-value this large could occur due to noise.

Fit statistics showed that the difference was less than 0.2, with adjusted R^2^ and predicted R^2^ values of 0.9598 and 0.8848, respectively. The adjusted R^2^ (0.9765) and projected R^2^ (0.8811) showed a minor difference (less than 0.2) that suggested the chosen model was adequate for response prediction. This validates the model’s ability to move across the design space efficiently.

The polynomial regression equation generated by the RSM for % EE with respect to factors A, B, and C is as follows:

% EE = +90.06 + 6.54* A − 2.39* B + 4.62* C − 0.2900* AB + 4.56* AC − 0.6750* BC + 0.8825* A^2^ − 5.65* B^2^ − 8.01* C^2^

where A is the molar ratio of E80 and DOPE, B is the molar concentration of CHEMS, and C is the amount of drug.

In the context of the aforementioned quadratic equation, the plus (+)and minus (–) signs indicate the direction of the change in the response, concerning the factors under examination. The 3D surface response plot, depicted in Figure 1G–I, distinctly shows a substantial increase in % EE that was concurrent with an elevation in the E80 and DOPE molar ratio. Similarly, raising the molar concentration of CHEMS and the quantity of drug resulted in a notable escalation in % EE, even if only up to a specific threshold. Subsequently, a non-linear decrease was observed upon further augmentation of the molar concentration of CHEMS and the drug.

#### 3.1.3. Impact of Independent Variables on pH Sensitivity (Response Y3)

As observed in Table 2, the % pH sensitivity (Y3) of liposomes, measured as % drug release at pH 5.5, ranged from 56.37 ± 1.36% to 90.14 ± 0.77%. The results of the one-way ANOVA for the quadratic model demonstrated a significant effect of the E80 and DOPE molar ratio, CHEMS molar concentration, and amount of drug on % pH sensitivity (*p*-value < 0.05). The lack of fit was deemed insignificant, with an F-value of 0.1130. In terms of fit statistics, the adjusted R^2^ and predicted R^2^ values were determined to be 0.9598 and 0.8248, respectively, indicating a difference of less than 0.2 and confirming the adequacy of the selected model for predicting responses.

The polynomial regression equation generated by RSM for % pH sensitivity with respect to factors A, B, and C is as follows:

% pH sensitivity = +81.25 + 8.61* A +5.04* B + 0.6263* C +1.91* AB −1.38* AC − 1.52* BC −5.12* A^2^ − 1.99* B^2^ − 8.43* C^2^

where A is the molar ratio of E80 and DOPE, B is the molar concentration of CHEMS, and C is the amount of drug.

In the given quadratic equation, both the plus (+) and minus (−) signs signify the direction of the change in the response relative to the factors. Among the selected independent variables and their interactions, A, B, A^2^, and C^2^ model terms were found to be significant (*p* < 0.05), suggesting a major contributing effect of the molar ratio of E80 and DOPE and the molar concentration of CHEMS on the % pH sensitivity of the liposomes. Increasing the molar ratio of E80 and DOPE and the molar concentration of CHEMS resulted in an increase in % pH sensitivity, as depicted in Figure 1J–L.

The investigation analyzed the standardized impact of the independent variables and their interactions on the dependent variable by generating a Pareto chart. This graphical representation delineates the primary effects of the independent variables and interactions, along with their respective significance levels on the dependent variables. The Pareto chart serves as a visual tool to prioritize and concentrate on the most influential factors for optimizing the process. The length of each bar in the chart reflects the standardized effect of that factor on the response.

**Figure 2:**
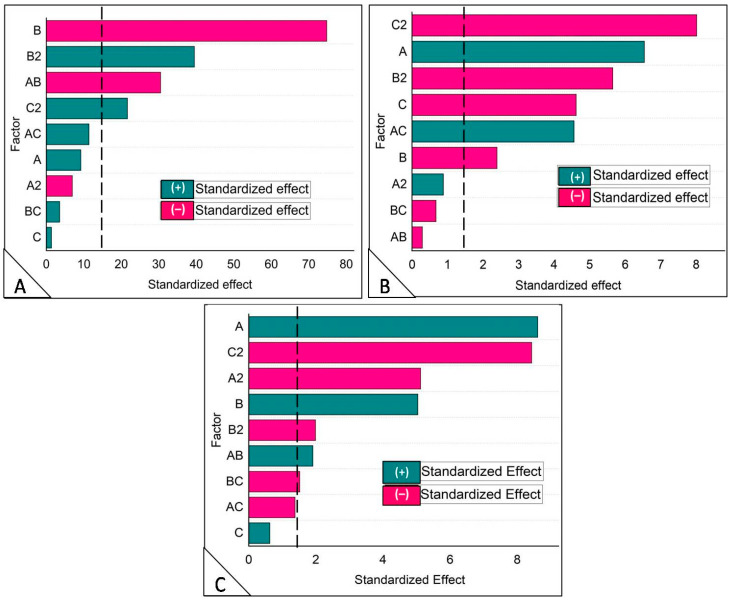
Pareto chart analysis of the Box-Behnken design showing the standardized effect of independent variables and their interaction on (**A**) PS, (**B**) % EE, and (**C**) % pH sensitivity. The vertical break black lines represent the threshold of significance (*p* = 0.05).

The impact of experimental factors on PS, % EE, and % pH sensitivity is illustrated in Figure 2A–C through a Pareto chart. Table 3 displays the polynomial coefficient estimate for the dependent variable and Table 4 displays the fit statistics of dependent variables (Regression coefficient). Factors whose length exceeds the line (*p*-value = 0.05) are deemed significant for the response value. The significance line values in Figure 2A–C were 14.96, 1.60, and 1.72, respectively.

#### 3.1.4. Search for Optimized Formulation and Point Prediction

**Figure 3:**
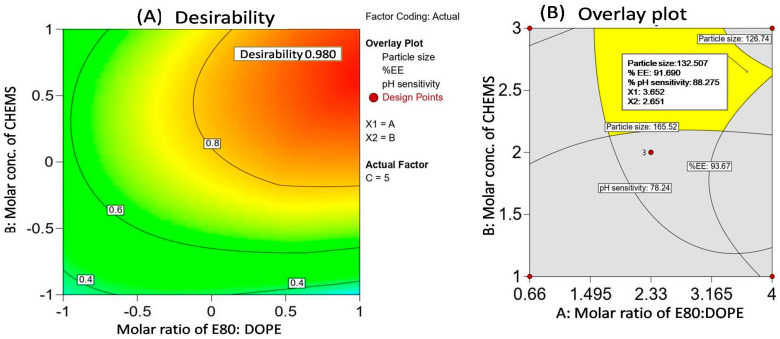
Desirability graph (**A**) and overlay plot (**B**) of optimized formulation.

**Figure 4:**
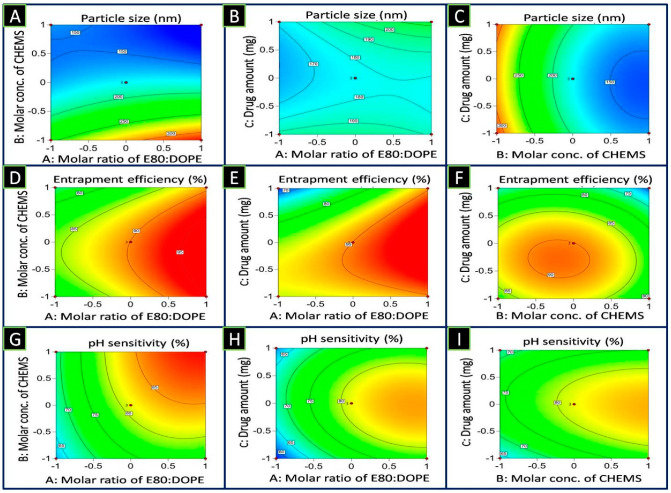
2D contour plots representing the impact of selected independent variables on dependent variables like particle size (**A**–**C**), % entrapment efficiency (**D**–**F**), and % pH sensitivity (**G**–**I**).

Optimization provides exact formulation parameters for the selected responses that accomplish the intended objectives. The numerical and graphical optimization technique allowed for optimization of the values of the independent variables for the 5-FU-pHLip formulation. For the purpose of numerical optimization, the responses and independent variables were set to the desired value. The range was established for independent variables A, B, and C. Y1 was the least answered, and Y2 and Y3 had the maximum responses. After calculating the expected values of the factors and desirability, it was found that the desirability value was 0.980, as seen in Figure 3A. The influence of independent variables on PS, % EE, and %pH sensitivity is shown in two-dimensional (2D) contour plot form in Figure 4.

The numerical standards for optimized 5-FU-pHLip were PS 126.7 ± 1.6 to 165.52 ± 1.8 nm, %EE 78.4 ± 1.6 to 93.7 ± 1.2%, and % pH sensitivity 78.2 ± 2.7 to 90.1 ± 0.8%. Based on this, the Box-Behnken design suggested the most suitable values for the formulation variables as a 3.65 molar ratio of E80 and DOPE, 2.65 molar concentration of CHEMS, and 5.18 amount of drug. From the overlay plot, as shown in Figure 3B, the predicted values for all the responses, such as PS (Y1), %EE (Y2), and % pH sensitivity (Y3), were found to be 132.5 nm, 91.7% and 88.3%, respectively.

### 3.2. Verification of Conjugates via FT-IR and ^1^H-NMR Spectroscopy

#### 3.2.1. DSPE-PEG_2000_-FA Conjugate

**Figure 5:**
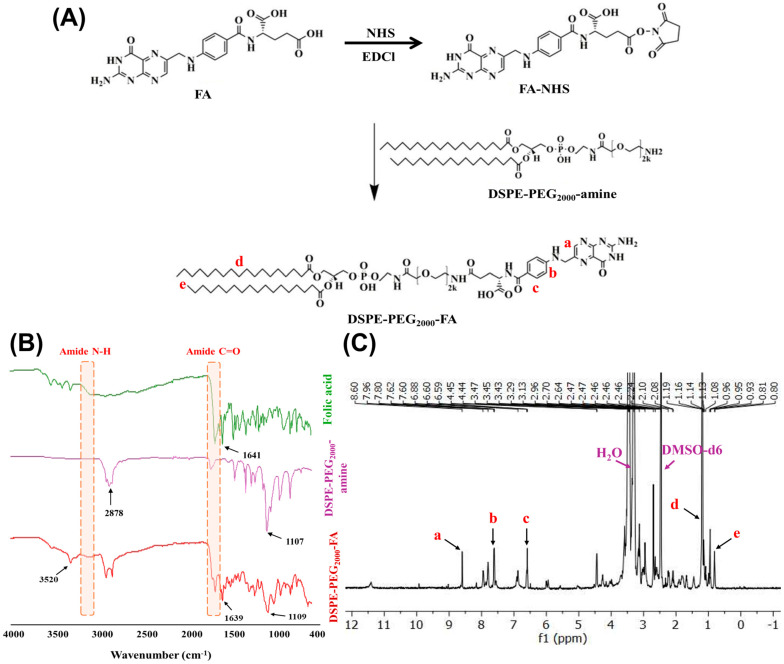
Proposed reaction scheme for DSPE-PEG_2000_-FA conjugate (**A**), FT-IR spectra of DSPE-PEG_2000_-FA conjugate (**B**), and ^1^H NMR spectra of DSPE-PEG_2000_-FA conjugate (**C**).

The DSPE-PEG_2000_-FA conjugate was synthesized according to the reaction shown in Figure 5A. The DSPE-PEG_2000_-FA with a yield of 74% was lyophilized and verified by spectroscopic techniques, including ^1^H-NMR and FT-IR spectroscopy.

Figure 5B represented the FT-IR spectra of DSPE-PEG_2000_-FA, the characteristic peaks of which were 3520 cm^−1^ and 3348 cm^−1^, respectively, ascribed to the N-H and O-H of FA. The peaks seen at 1639 cm^−1^ are indicative of the presence of aromatic C=C double bonds in FA. The presence of characteristic peaks corresponding to amide N-H stretching and C=O stretching at 3239 cm^−1^ and 1742 cm^−1^, respectively, indicate the formation of amide II bonds. Moreover, the vibrations at 1109 cm^−1^ showed PEG C-O stretching of DSPE-PEG_2000_-amine and CH_2_ stretching of DSPE at 2894 cm^−1^ confirming the synthesis of DSPE-PEG_2000_-FA.

Figure 5C represents the ^1^HNMR spectra of the DSPE-PEG_2000_-FA conjugate in which the *p*-phenyl ring and pteridine ring showed the chemical shift of 8.6 (a), 7.6 (b), and 6.6 (c) ppm. The spectra exhibit distinct peaks at 7.96 ppm, indicating the presence of the amide linkage between DSPE-PEG_2000_-amine and FA. The characteristic peaks at 2.46 ppm and 3.29 ppm were also recognized as the DMSO-d6 and water separately. The synthesis of DSPE-PEG_2000_-FA was validated by the chemical shifts at 0.8 (d) and 1.19 (e) ppm, which correspond to the proton peaks of the methyl and methylene groups of the DSPE-PEG_2000_-amine, respectively.

#### 3.2.2. DSPE-PEG_2000_-iRGD Conjugate

**Figure 6:**
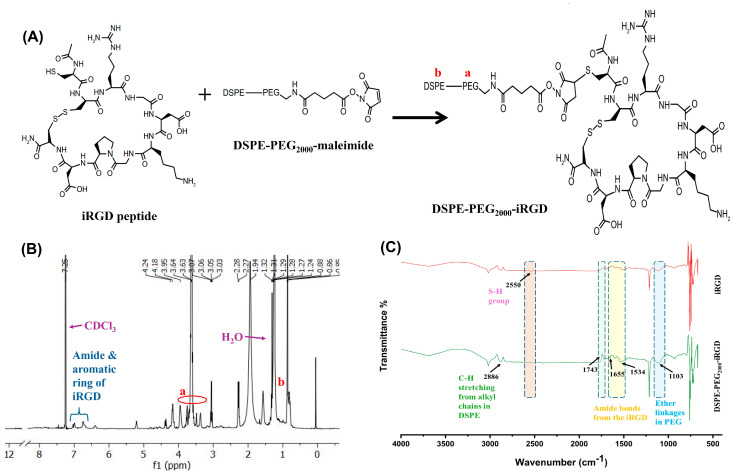
Proposed reaction scheme for DSPE-PEG_2000_-iRGD (**A**); ^1^H NMR spectra of DSPE-PEG_2000_-iRGD conjugate (**B**); and FT-IR spectra of DSPE-PEG_2000_-iRGD (**C**).

The DSPE-PEG_2000_-iRGD was synthesized according to the reaction shown in Figure 6A. The prepared conjugate with a yield of 86% was further lyophilized and analyzed using ^1^HNMR and FT-IR spectroscopy to confirm the conjugation of iRGD and DSPE-PEG_2000_-Mal. The ^1^HNMR spectrum of DSPE-PEG_2000_-iRGD was performed in chloroform solvent, as depicted in Figure 6B. The characteristic peaks at 1.57 ppm and 7.25 ppm were recognized as water and CDCl_3,_ respectively_._ The presence of the peaks in the 3.4–4.0 ppm (a) range confirms the PEG chains, while peaks in the 1.0–2.5 ppm (b) range are indicative of the DSPE aliphatic chains. The downfield peaks at δ ~6.5–7.0 ppm confirm the presence of the protons from amide bonds and aromatic rings in iRGD peptide. The chemical shift of the thiol group in DSPE-PEG-Mal and a peak of iRGD would typically appear in the region between 2.5 to 3.0 ppm and 7.0–7.2 ppm, but here it is not detected, which confirms the thiol group and cysteine group of the iRGD peptide formed a thioether linkage during Michael additive reaction. The FT-IR spectra (Figure 6C) showed the peak at 2886 cm^−1^ indicates strong C-H stretching from alkyl chains in DSPE. The peak at 1103 cm^−1^ is a broad peak confirming the ether linkages in PEG. The broad peak at 1534 cm^−1^ and the peak at 1655 cm^−1^ indicate the amide bonds from the iRGD peptide. The -SH group is observed at 2550 cm^−1^ in the iRGD spectra but is absent in the DSPE-PEG_2000_-iRGD spectra. The peak has shifted and an inverted peak around 1700–1750 cm^−1^ confirms the successful conjugation of the maleimide group with the thiol (-SH) group of iRGD in the DSPE-PEG_2000_-iRGD spectra.

### 3.3. Characterization of the Prepared 5-FU-pHLip and FA-iRGD-5-FU-pHLip

#### 3.3.1. Particle Size (PS), Polydispersity Index (PDI), and Zeta Potential (ZP)

After implementing the Box-Behnken Design (BBD) experimental approach, the predicted PS (D_H_) of the optimized pHLip was determined to be 132.50 ± 3.2 nm, while the actual size (D_H_) obtained was 139.7 ± 2.1 nm, showing a slight deviation from the predicted value. A comprehensive analysis of the correlation between particle size and the three aforementioned factors (A, B, and C) was already provided in the experimental design and optimization section.

Regarding the PDI, the optimized pHLip and formulated FA-iRGD-5-FU-pHLip exhibited values of 0.26 ± 0.1 and 0.22 ± 0.2, respectively, indicating uniform and well-dispersed liposomal formulations. The zeta potentials ZPs of 5-FU-pHLip and FA-iRGD-5-FU-pHLip were measured to be −12.2 ± 3.6 mV and −14.8 ± 2.8 mV, respectively. The zeta potential of both pHLip and surface-modified pHLip were found to be close to neutral, likely attributed to the inclusion of DSPE-PEG_2000_ within the liposome lipid composition. This incorporation of PEG chains onto the liposome surface creates a hydrophilic layer, resulting in a reducing effect on the electrostatic attraction between DOPE and H^+^ ions. Additionally, PEG chains play a vital role in preventing vesicle aggregation and fusion, enhancing stability, and facilitating prolonged circulation in the body.

#### 3.3.2. Morphological Characterization by TEM and AFM

**Figure 7:**
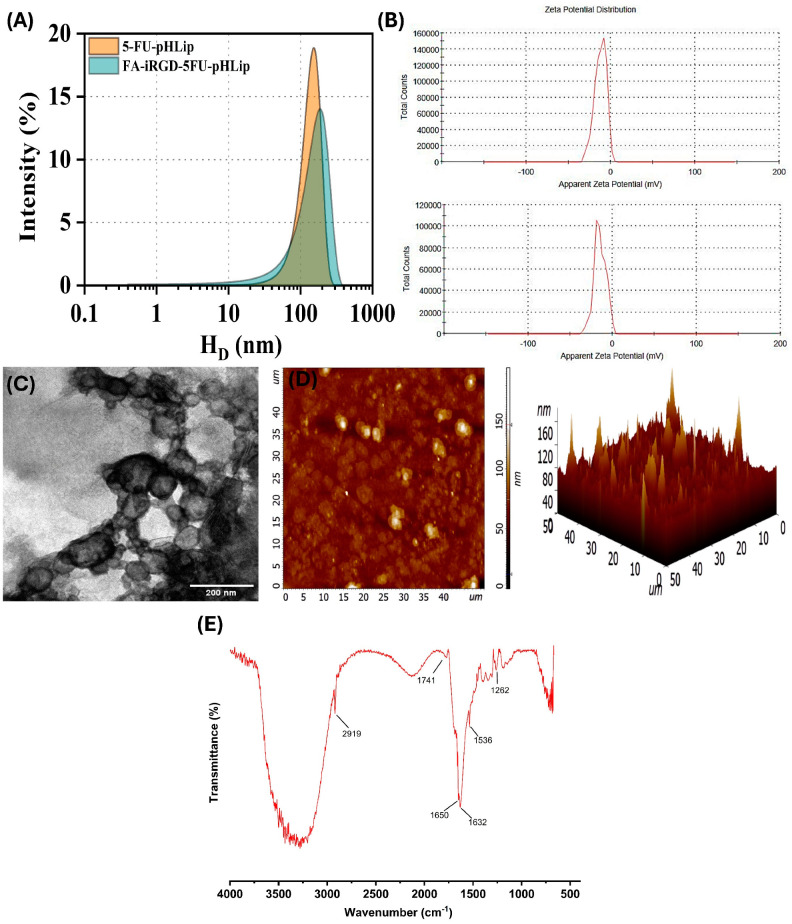
Particle size intensity distribution (**A**) and zeta potential (**B**) of 5-FU-pHLip and FA-iRGD-5-FU-pHLip, respectively. TEM (**C**), AFM (**D**), and (**E**) FT-IR micrographs of FA-iRGD-5-FU-pHLip.

Figure 7C,D displays the results of the TEM and AFM used to measure the morphology and shape of the developed pH-Lip. As seen in the TEM image, the particle size ranges from 120 ± 2.6 to 150 ± 4.2 nm. The AFM micrographs clearly show that the majority of the particles have sizes close to 150 nm, with the formulation exhibiting almost spherical shapes. These observations correlate with the sizes (D_H_) obtained from the DLS analysis, which is 152 nm.

#### 3.3.3. FTIR Analysis

The FTIR spectroscopy analysis of the FA-iRGD-5-FU pH-sensitive liposomes revealed several key functional groups, confirming the successful incorporation of the formulation components (Figure 7E). A peak observed at approximately 3400 cm⁻¹ is indicative of O-H stretching vibrations, which likely originate from the hydroxyl groups in the PEG chains of DSPE-PEG_2000_ and the carboxyl groups in FA. Additionally, the presence of peptide bonds in iRGD contributes to this absorption band. The peak at 2919 cm⁻¹ corresponds to C-H stretching vibrations, confirming the inclusion of aliphatic chains from the lipid components (PC, DOPE, and CHEMS) and alkyl groups in DSPE. The peak at 1741 cm⁻¹, attributed to C=O stretching vibrations, signifies the presence of carbonyl groups from the ester bonds of the lipids and the carbonyl group of 5-Fluorouracil. Furthermore, the peaks at 1650 cm⁻¹ and 1536 cm⁻¹ correspond to the amide I and amide II bands, respectively, indicating C=O stretching and N-H bending/C-N stretching vibrations from peptide bonds in iRGD and amide linkages in DSPE-PEG_2000_-FA. Peaks observed in the 1000–1150 cm⁻¹ region, indicative of C-O-C stretching vibrations, confirm the presence of PEG chains in the formulation.

#### 3.3.4. Entrapment Efficiency and Drug Loading

**Figure 8:**
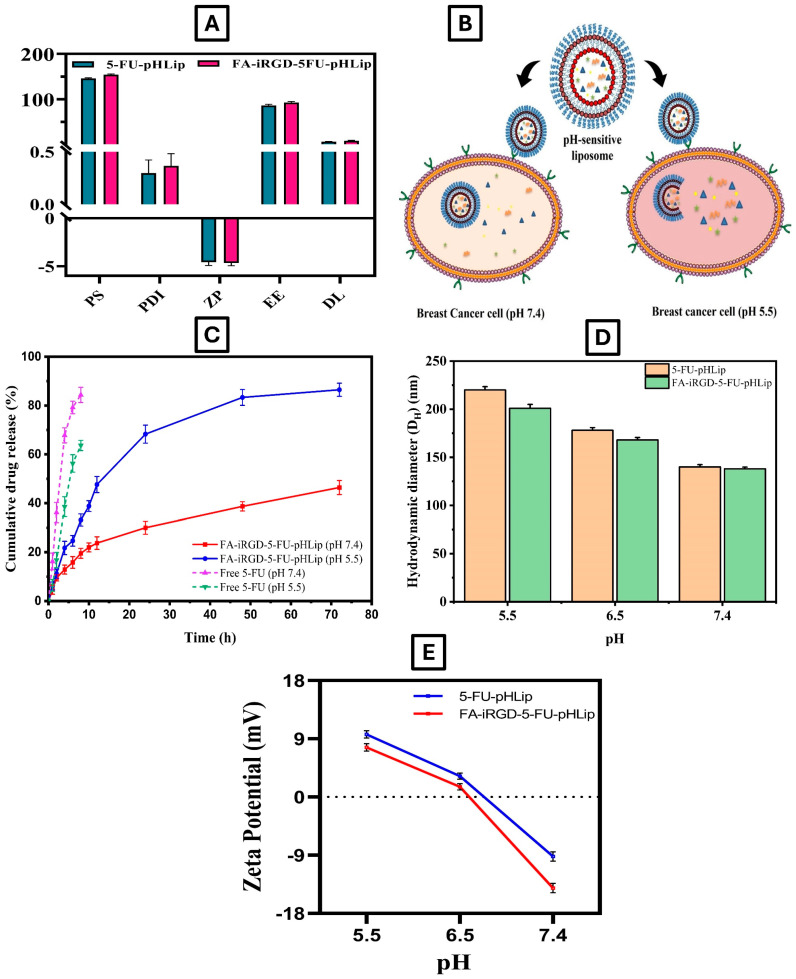
Physico-chemical characterization of the developed pH-sensitive liposomes (**A**), possible scenario of the pH sensitivity of FA-iRGD-5-FU-pHLip at pH 7.4 and 5.5 (**B**), in-vitro % pH sensitivity of free 5-FU drug and FA-iRGD-5-FU-pHLip at pH 7.4 and 5.5 (**C**), pH-induced liposomal aggregation assays of 5-FU-pHLip and FA-iRGD-5-FU-pHLip formulations in different pH media (**D**,**E**).

According to the findings, the %EE of 5-FU in 5-FU-pHLip & FA-iRGD-5-FU-pHLip were determined to be 88.9 ± 2.8 and 93.1 ± 2.6%, respectively. The predicted %EE of 5-FU-pHLip was 91.69, deviating marginally from the observed %EE value. Similarly, the observed values of %DL of 5-FU-pHLip and FA-iRGD-5-FU-pHLip were 6.0 ± 2.4 and 7.4 ± 2.8%, respectively. The drug loadings in µg drug/mg lipid for 5-FU-pHLip and FA-iRGD-5-FU-pHLip were calculated to be 60.3 µg/mg and 74.1 µg/mg, respectively. A summary of the observed values for each physicochemical parameter was provided in the graph (Figure 8A).

#### 3.3.5. In-Vitro pH-Sensitive Drug Release Study

The pH-sensitive drug release of the free drug and developed FA-iRGD-5-FU-pHLip was assessed in-vitro at various pH values (7.4 and 5.5) for a period of 72 h. PBS solution with a pH of 7.4 replicates the surroundings of the systemic circulation, whereas a pH of 5.5 replicates the acidic pH found in endo-lysosomes. A possible scenario of the pH sensitivity of FA-iRGD-5-FU-pHLip at pH 7.4 and 5.5 was shown in Figure 8B. The outcomes of this study are illustrated in Figure 8C, which demonstrated that FA-iRGD-5-FU-pHLip exhibited a rapid response to acidic pH. At pH 5.5, the release percentages of 5-FU from FA-iRGD-5-FU-pHLip were 68.3 ± 3.7%, 83.3 ± 4.3%, and 84.5 ± 6.2% at 24, 48, and 72 h, respectively. In contrast, at pH 7.4, the release percentages were only 30.0 ± 3.7%, 38.7 ± 3.9%, and 46.4 ± 5.7% at the same time points. These findings indicate that the liposomes remain stable in physiological conditions (pH 7.4), suggesting potential resistance to blood circulation changes and the maintenance of formulation integrity until reaching the therapeutic target. Conversely, in acidic conditions (pH 5.5), the pH sensitivity of the liposomes was clearly demonstrated by alterations in the supramolecular arrangement of DOPE molecules under high water content conditions. This alteration is critical for facilitating the delivery of 5-FU into the cytoplasm of targeted cancer cells, potentially enhancing the drug’s concentration and antitumor activity.

### 3.4. pH-Induced Liposomal Aggregation Assay

A study on pH-induced flocculation in buffers with different pH values showed that 5-FU in pH-sensitive liposomal formulations is stable in neutral buffers but flocculates and releases quickly at lower pH values (i.e., the acidic range). The pH-dependent variations in PS and ZP were used to measure the liposomal formulations’ fusogenicity. The outcomes of pH-induced liposomal aggregation in assays of 5-FU-pHLip and FA-iRGD-5-FU-pHLip formulations in different pH media were displayed in Figure 8D.

The increase in the D_H_ of FA-iRGD-5-FU-pHLip with decreasing pH is due to the behavior of DOPE, a critical component of pH-sensitive liposomes, which undergoes a phase transition from a lamellar to an inverted hexagonal phase in acidic conditions, leading to membrane fusion and aggregation. This change is irreversible upon returning to neutral pH, indicating permanent structural alterations. Such pH-induced fusion enhances drug delivery in the acidic tumor microenvironment by promoting effective drug release, while maintaining stability while in circulation until the target site is reached. This ensures that the drug is not prematurely released, enhancing its therapeutic efficacy and stability in biological systems.

Furthermore, zeta potential measurements were conducted in various pH buffer conditions. For the FA-iRGD-5-FU-pHLip, the ZP shifted from −14.2 ± 1.2 mV at pH 7.4 (physiological alkaline) to 7.4 ± 1.2 mV at pH 5.5 (endosomal acidic).

The irreversible increase in particle size at lower pH levels indicates that the liposomal membranes undergo fusion when exposed to acidic conditions. This fusogenic behavior is likely due to the structural changes in the liposomal membrane components in response to pH changes, promoting lipid mixing and fusion. The shift in the ZP towards a positive value at acidic pH further supports this, as the protonation of liposomal surface groups reduces electrostatic repulsion and facilitates closer interaction between the liposomes.

### 3.5. In-Vitro Biological Stability Assessment

**Figure 9:**
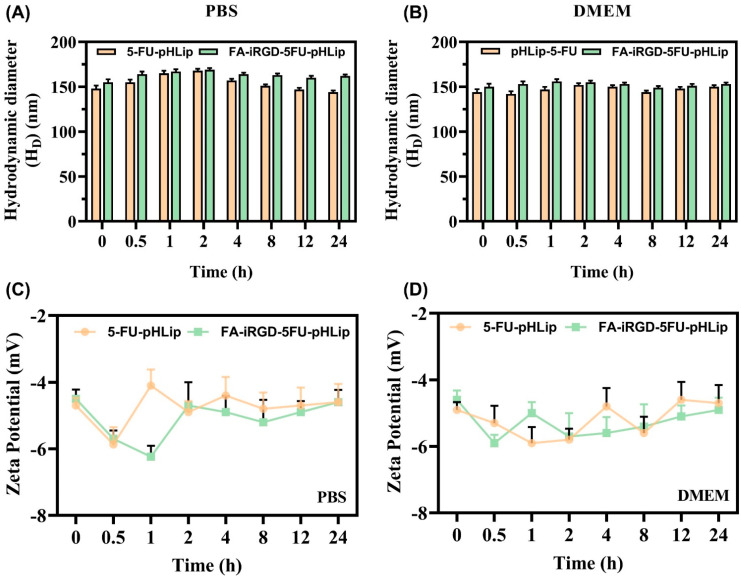
In-vitro stability of liposomes under study in PBS (pH 7.4) and DMEM in terms of mean particle size (**A**,**B**) and zeta potential (**C**,**D**), respectively. Data are expressed by the mean (*n* = 3) ± SD.

The developed pH-sensitive liposome was incubated in PBS (pH 7.4) and in a cell culture medium (DMEM) for 24 h, and after that, the mean PS (D_H_) and ZP were measured. A 10% (*v*/*v*) FBS supplement was added to each medium. As shown in Figure 9, irrespective of the media, 5-FU-pHLip and FA-iRGD-5-FU-pHLip demonstrated appropriate stability. There were no obvious changes in the mean diameter or ZP (*p* > 0.05).

### 3.6. Serum Stability Studies

**Figure 10:**
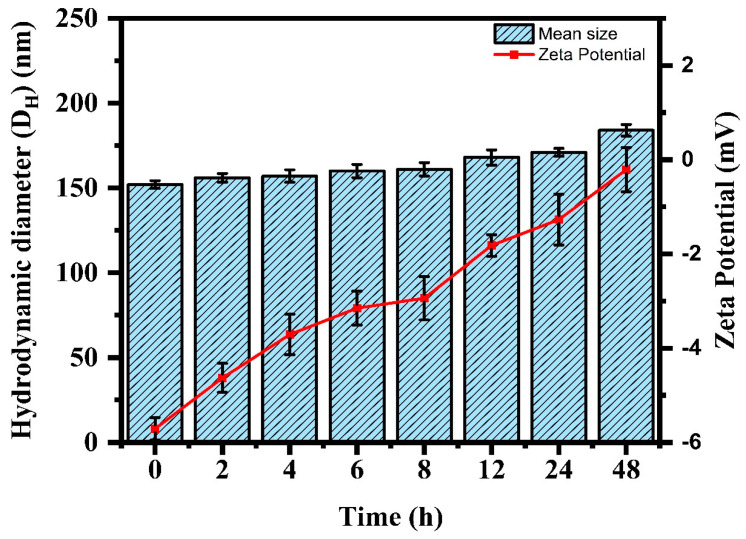
Serum stability assessment data of the developed FA-iRGD-5-FU-pHLip in terms of mean particle size and zeta potential.

For the FA-iRGD-5-FU-pHLip, a gradual increase in the D_H_ of liposomes was observed from 152.2 ± 2.3 nm to 184.2 ± 3.8 nm by the end of the 48 h incubation period in the presence of serum (Figure 10). Concurrently, changes in the zeta potential were also measured and results revealed that the initial zeta potential of −5.7 ± 0.3 mV shifted to −0.21 ± 0.5 mV after 48 h of incubation in serum (Figure 10). The observed increase in particle size and the changes in the zeta potential are attributed to the interaction with serum proteins. When liposomes are stored in serum, proteins in the serum can adsorb onto the liposomes’ surfaces and change the surface characteristics of the liposomes. The shift towards a less negative zeta potential indicates a reduction in the electrostatic repulsion between particles, further promoting aggregation.

### 3.7. Storage Stability Studies

**Figure 11:**
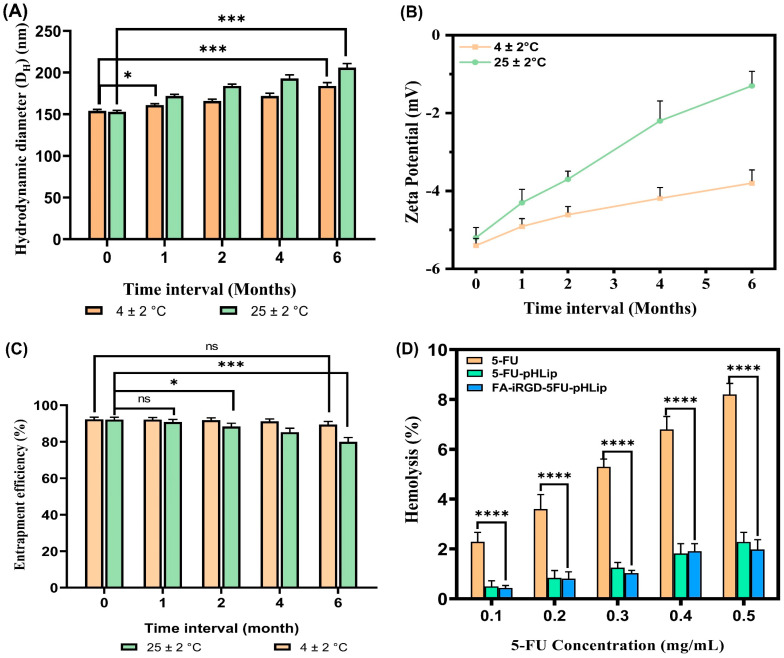
Storage stability data in terms of the mean particle size (**A**), zeta potential (**B**), and entrapment efficiency (**C**) up to 6 months: % hemolysis plots of plain 5-FU solutions and liposomal formulations at various 5-FU concentrations (**D**). Data are expressed by the mean (*n* = 3) ± SD. [One asterisk (*), three asterisks (***), and four asterisks (****) denote significant *p* values, i.e., <0.05, <0.001 and <0.0001, respectively, and ns as non-significant].

The D_H_ of the liposomal formulations stored at a refrigerated temperature (4 ± 2 °C) and room temperature (25 ± 2 °C) was monitored at regular intervals. The particle size increased gradually over time, likely due to particle fusion or agglomeration, as shown in Figure 11A. This increase was more pronounced at 25 ± 2 °C compared to 4 ± 2 °C. Specifically, the mean particle diameter of the pHLip liposomal suspension stored at 4 ± 2 °C increased from an initial 154.7 ± 1.9 nm to 184.61 ± 3.8 nm, while the sample stored at 25 ± 2 °C increased to 206.4 ± 4.8 nm after 6 months.

The zeta potential of the liposomal formulation remained close to neutral. At 4 ± 2 °C, the zeta potential decreased from −5.4 ± 0.2 mV to −3.8 ± 0.3 mV, whereas at 25 ± 2 °C, it decreased further to −1.4 ± 0.4 mV (Figure 11B).

Entrapment efficiency (%EE) is a critical parameter for assessing the stability of liposomal preparations in order to evaluate the potential for drug leakage during storage. The results (Figure 11C) revealed that the %EE of the formulation stored at 4 ± 2 °C decreased slightly from 93.1 ± 2.6% to 89.5 ± 1.7%. In contrast, the sample stored at 25 ± 2 °C showed a more significant decrease to 80.0 ± 2.3% after 6 months.

These results suggest that pHLip formulations stored at higher temperatures exhibit less stable profiles, underscoring the importance of appropriate storage conditions. Storing liposomal formulations at lower temperatures or using lyophilization (freeze drying) techniques can help maintain their integrity and stability over extended periods. The observed increases in particle size and decreases in the %EE and zeta potential at higher temperatures highlight the need for proper storage to preserve the formulation’s therapeutic efficacy.

### 3.8. Hemolysis Study

To ensure the safest possible intravenous (i.v.) administration, the hemolytic potential of the liposomal formulations was studied. Hemolysis indicates irritation of RBCs by the formulation, a critical issue for reliable in-vivo drug delivery. The results showed that the hemolytic behavior of the pure drug is dose-dependent, with higher concentrations causing greater hemolysis. The experiment used PBS as a negative control and Triton-X as a positive control to indicate complete hemolysis. The surface-decorated pHLip formulation exhibited a modest hemolytic potential compared to the free drug at various concentrations (0.1–0.5 mg/mL), as illustrated in Figure 11D. The hemolytic data revealed significantly lower hemolysis (*p* < 0.05) for the liposomal formulation compared to the naïve drug at all tested concentrations. Liposomal formulations have been able to decrease the hemolytic activity of 5-FU, perhaps due to slower drug release, especially at pH 7.4.

The modest hemolytic potential observed with the surface-decorated pHLip formulation suggests that it interacts less with the RBC membrane, reducing the risk of hemolysis and making it a safer option for intravenous administration.

### 3.9. Wound Healing by Scratch Assay

**Figure 12:**
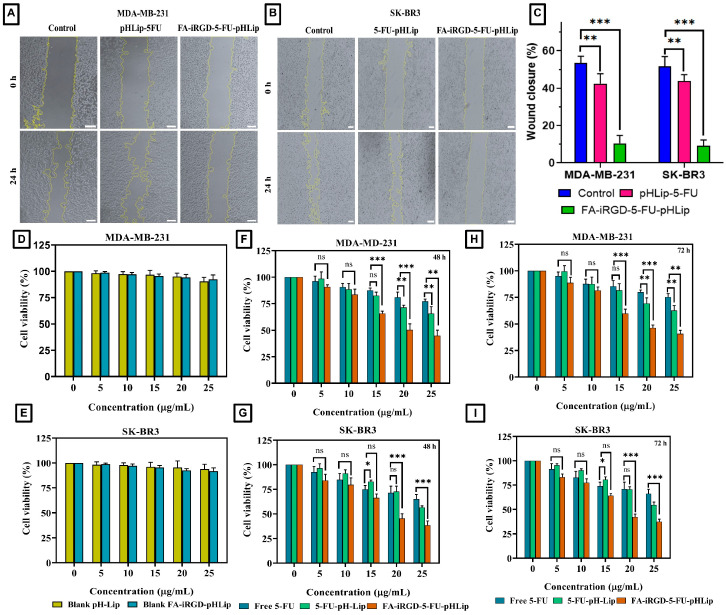
Wound healing assay (**A**,**B**); the % of wound closure plot (**C**); viable MDA-MB-231 and SK-BR3 cell percentages versus an empty liposome concentration equivalent to that used for drug-loaded liposomes in Figures (**F**,**G**) over the period of 72 h (**D**,**E**); viable MDA-MB-231 and SK-BR3 cell percentage versus concentrations of 5-FU in cells treated with free 5-FU, 5-FU-pHLip, and FA-iRGD-5-FU-pHLip over the period of 48 h (**F**,**G**) and 72 h (**H**,**I**), respectively. [One asterisk (*), two asterisks (**), and three asterisks (***) denote significant *p* values, i.e., <0.05, <0.01, and <0.001, respectively, and ns as non-significant].

Wound healing assays are commonly used to evaluate the impact of drug treatments on cell motility, which is crucial in cancer research for understanding the metastasis process potential. Treatments that effectively inhibit cell motility are highly desirable, as they could potentially reduce metastasis. For the MDA-MB-231 cell line, the FA-iRGD-5-FU-pHLip demonstrated significant efficacy in inhibiting cell motility (Figure 12A). After 24 h, only 10.50 ± 4.22% of the wound area was healed when treated with the surface-decorated pHLip formulation, compared to cells treated with the pure drug and 5-FU-pHLip, as shown in Figure 12C. This suggests that the surface-decorated formulation is more effective in inhibiting cell migration. Similarly, in SK-BR3 cells, the FA-iRGD-5-FU-pHLip formulation also showed pronounced inhibition of cell motility. This behavior underscores the potential of the surface-decorated pHLip formulation to impede the metastatic spread of cancer cells more effectively than the pure drug alone. The enhanced inhibition of cell motility by the FA-iRGD-5-FU-pHLip formulation can be attributed to several factors. The surface decoration likely improves the targeting and uptake of the liposomes by the cancer cells, enhancing the delivery of the encapsulated drug. Additionally, the pHLip modification aids in the selective release of the drug in the acidic tumor microenvironment, increasing its local concentration and effectiveness. These combined effects result in a more pronounced inhibition of cell motility, making the surface-decorated pHLip formulation a promising candidate for anti-metastatic cancer therapy.

### 3.10. Cytotoxicity Study

Cell cytotoxicity was assessed to compare the efficacy of the developed formulations, including blank pHLip, blank FA-iRGD-pHLip liposomes, free 5-FU, 5-FU-pHLip, and FA-iRGD-5-FU-pHLip, after a 48 & 72 h exposure.

Figure 12D–G, demonstrate the results of analyzing the concentration-dependent cytotoxicity profiles of these formulations in the MDA-MB-231 and SK-BR3 cell lines. All experiments were repeated three times for accuracy. No significant cytotoxicity was reported at the highest concentration for blank liposomes. However, both 5-FU-pHLip and FA-iRGD-5-FU-pHLip showed significantly higher (*p* < 0.001) cytotoxicity than free 5-FU at 15, 20, and 25 μg/mL against the MDA-MB-231 (Figure 12F). On the other hand, both 5-FU-pHLip and FA-iRGD-5-FU-pHLip showed significantly higher (*p* < 0.001) cytotoxicity than free 5-FU at only 20 and 25 μg/mL against the SK-BR3 cells (Figure 12G). Moreover, the obtained results revealed the cell viability percentage for free 5-FU, 5-FU-pHLip, and FA-iRGD-5-FU-pHLip treatments to be 77.1 ± 2.1%, 65.5 ± 6.7%, and 44.8 ± 5.3% in 48 h, respectively, and 75.2 ± 3.3%, 62.5 ± 4.7, and 40.8 ± 3.3%, respectively, against the MDA-MB-231 after 72 h. Whereas, SK-BR3 cells showed viable cell percentages of 65.1 ± 4.1%, 56.1 ± 1.5%, and 38.4 ± 4.5% at 48 h, and 66.1 ± 4.5, 54.6 ± 3.0., and 37.40 ± 2.5 following treatment with free 5-FU, 5-FU-pHLip, and FA-iRGD-5-FU-pHLip, after 72 h, respectively. The lack of significant differences in cell viability between 48 and 72 h suggests that the maximum cytotoxic effect of the formulations may have been achieved within the first 48 h. This plateau in cytotoxicity could indicate that most of the susceptible cells have been affected by 48 h. It is also possible that the remaining viable cells at 48 h exhibit some degree of resistance to further cytotoxic effects, leading to similar viability percentages at 72 h.

The observed higher cell viability for free 5-FU compared to 5-FU-pHLip and FA-iRGD-5-FU-pHLip can be attributed to the differences in the drug delivery mechanisms. Free 5-FU relies on passive diffusion into cells, which might be less efficient compared to the targeted delivery offered by the liposomal formulations. FA-iRGD-5-FU-pHLip enhanced cellular uptake through receptor-mediated endocytosis, facilitated by the targeting ligands (FA and iRGD). This targeted delivery ensures a higher intracellular concentration of 5-FU, resulting in enhanced cytotoxicity and lower cell viability.

## 4. Conclusions

In this research work, we successfully developed, optimized, and characterized 5-FU-loaded, pH-sensitive liposomes (5-FU-pHLips).

*Novel formulation:* By incorporating DSPE-PEG_2000_-FA and DSPE-PEG_2000_-iRGD conjugates, we created a novel dual surface-modified liposomal formulation of 5-FU (FA-iRGD-5-FU-pHLip).

*pH responsiveness:* This formulation demonstrated pH responsiveness, with enhanced drug release at an acidic pH 5.5 compared to a physiological pH 7.4.

*Enhanced specificity and efficacy:* The novelty of this research lies in the dual-targeting mechanism utilizing FA and iRGD peptides, combined with the pH-sensitive properties of the liposomes, to enhance selective targeting and uptake by cancer cells and effective drug release in the acidic tumor environment.

*Shelf stability and integrity:* The FA-iRGD-5-FU-pHLip exhibited long-term shelf stability and maintained integrity under in-vitro conditions in media mimicking that of biological fluids.

*Improved cytotoxicity:* Notably, the FA-iRGD-5-FU-pHLip showed better cytotoxicity in comparison to the 5-FU-pHLip and free 5-FU against the MDA-MB-231 and SK-BR3 cell lines.

*Preclinical potential:* These promising results support the further preclinical development of FA-iRGD-5-FU-pHLip, representing a significant advancement in targeted cancer therapy.

## Figures and Tables

**Table 1:** Defining different variables and their respective Box-Behnken design levels for optimizing pHLip formulation.

Variables	Levels		
	−1	0	+1
***Independent variables***			
A = Molar ratio of E80:DOPE	2:3	3:2	4:1
B = Molar conc. of CHEMS	1	2	3
C = Drug amount (mg)	2.5	5	7.5
***Dependent variables***	***Constraints***		
Y1 = Particle size (nm)	Minimum		
Y2 = Entrapment efficiency (%)	Maximum Maximum		
Y3 = pH sensitivity (%)			

**Table 2:** Box-Behnken experimental design (BBD) presenting the outcome of independent variables on dependent variables.

Independent Variable				Dependent Variable (Response)		
Formulation Code	Factor 1 (A)	Factor 2 (B)	Factor 3 (C)	PS (nm) Y1	EE (%) Y2	pH Sensitivity (%) Y3
pHLip1	1	0	1	202.5 ± 1.3	89.3 ± 2.0	76.3 ± 0.5
pHLip 2	−1	0	1	170.3 ± 0.8	66.0 ± 2.2	60.7 ± 1.1
pHLip 3	0	1	−1	143.2 ± 1.2	78.4 ± 1.6	76.0 ± 2.4
pHLip 4	0	0	0	165.5 ± 1.8	90.4 ± 2.0	78.2 ± 2.6
pHLip 5	−1	1	0	160.3 ± 1.9	77.5 ± 0.8	70.2 ± 1.90
pHLip 6	−1	0	−1	204.1 ± 0.9	85.6 ± 1.1	56.4 ± 1.3
pHLip 7	0	1	1	166.9 ± 1.2	69.2 ± 1.2	73.8 ± 2.0
pHLip 8	1	1	0	126.7 ± 1.5	88.7 ± 1.6	90.1 ± 0.8
pHLip 9	−1	−1	0	231.8 ± 2.1	81.3 ± 0.6	61.9 ± 1.3
pHLip 10	0	−1	1	326.4 ± 1.2	75.7 ± 1.5	68.7 ± 1.7
pHLip 11	1	−1	0	320.2 ± 1.3	93.7 ± 1.1	74.3 ± 1.5
pHLip 12	0	0	0	177.7 ± 0.3	89.2 ± 1.8	87.6 ± 0.4
pHLip 13	0	−1	−1	316.9 ± 1.3	82.3 ± 0.5	64.8 ± 1.4
pHLip 14	0	0	0	188.5 ± 1.7	90.6 ± 1.4	78.0 ± 1.7
pHLip 15	1	0	−1	190.8 ± 2.4	90.8 ± 1.6	77.4 ± 1.2

**Table 3:** Generated model summary statistics of all responses measured as per BBD.

Polynomial Coefficients Estimates for Dependent Variables			
Factor	Particle Size (nm)	Entrapment Efficiency (%)	pH Sensitivity (%)
A	+9.2	+6.5	+8.6
B	−74.8	−2.4	+5.0
C	+1.4	−4.6	+0.6
AB	−30.5	−0.3	+2.0
AC	+11.4	+4.5	−1.4
BC	+3.6	−0.7	−1.5
A^2^	−7.0	+0.9	−5.1
B^2^	+39.5	−5.7	−2.0
C^2^	+21.6	−8.0	−8.4

**Table 4:** Fit statistics of dependent variables (responses).

Dependent Variable (Response)	Regression Coefficient						
	Std. Dev.	Mean	% CV	R^2^	Adjusted R^2^	Predicted R^2^	Adequate Precision
PS (nm)	16.68	206.12	8.09	0.9763	0.9336	0.8822	15.4566
EE (%)	1.29	83.25	1.55	0.9916	0.9765	0.8818	25.8535
pH sensitivity (%)	3.75	72.96	5.14	0.9433	0.8412	0.7594	10.6428

## Data Availability

The original contributions presented in the study are included in the article; further inquiries can be directed to the corresponding author.
